# Temporal Trends and Future Projections of P ulmonary Embolism and Top Contributing Causes of death in the United States, 1968–2040: insights from the CDC WONDER database

**DOI:** 10.1007/s11239-026-03281-9

**Published:** 2026-05-24

**Authors:** Saifullah Khan, Faizan Abbas, Muhammad Hassan, Muhammad Hussain, Muhammad Ahmed, Aiza Ahsan, Shaheer Qureshi, Nisha Khalid, Asim Sajjad, Maria Baig, Saad Ahmed Waqas, Gregg C. Fonarow, Stephen J. Greene, Faizan Ahmed

**Affiliations:** 1 Department of Dow Medical College, Dow University of Health Sciences, Karachi, Pakistan; 2 Department of Medicine, Fatima Memorial Hospital, Lahore, Pakistan; 3 Ahmanson-UCLA Cardiomyopathy Center, Ronald Reagan-UCLA Medical Center, Los Angeles, CA USA; 4 Division of Cardiology, Duke University School of Medicine, Durham, NC USA; 5 Duke Clinical Research Institute, Duke University School of Medicine, Durham, NC USA; 6 Hackensack Meridian Health Jersey Shore University Medical Center, Neptune, USA

**Keywords:** Pulmonary embolism, CDC WONDER, Auto-ARIMA, Prophet model, Annual percent change, Epidemiology, Aging population, Cardiovascular risk factors

## Abstract

Pulmonary embolism (PE) is a preventable yet often deadly cardiovascular event. We aimed to investigate PE mortality trends from 1968–2023 and forecast rates through 2040 in the U.S. We analyzed CDC WONDER death certificates for adults ≥ 25 years. Age-adjusted mortality rates (AAMRs), annual percent changes (APCs) were used to analyse trends and Auto-ARIMA/Prophet forecasting models were used for future projections, all stratified by sex, age, race, and contributing cause of death. From 1968–2023, 516,187 PE deaths occurred, with a mean AAMR of 6.06 per 100,000 (AAPC -1.85). Mean AAMRs were 6.76 in men and 5.57 in women. By age, rates averaged 0.98 for adults 25–44 years, 3.96 for 45–64 years, and 21.73 for ≥ 65 years. Black adults had a mean AAMR of 10.87 versus 5.76 in White adults. From 1999–2023, PE death linked to Coronary Artery Disease, COPD, infection, and diabetes declined, while PE death linked to hypertension rose and heart failure related PE plateaued. Forecasts project an overall AAMR of 3.63 by 2040 (2.32 in men, 3.25 in women), with the highest predicted rate of 9.24 among adults ≥ 65 years. Projected racial AAMRs are 3.70 in White and 7.71 in Black individuals. Hypertension and heart failure linked rates are expected to rise further. Despite a sustained decline in PE death since 1968, persistent racial gaps, aging populations, and rising hypertension and heart-failure related PE threaten progress, highlighting the need for targeted prevention and equitable cardiovascular risk management.

**Supplementary Information:**

The online version contains supplementary material available at https://doi.org/10.1007/s11239-026-03281-9.

## Introduction

Pulmonary embolism (PE), the most severe form of venous thromboembolism (VTE), continues to be a leading preventable cause of cardiovascular death [1, 2]. It originates by thrombus migration into the pulmonary arteries, typically from the deep veins of the lower limbs, resulting in diminished gas exchange, right ventricular strain, and hemodynamic compromise [2]. Despite major diagnostic and therapeutic advances, PE continues to cause an estimated 100,000 deaths annually in the United States, ranking as a leading cause of cardiovascular mortality after myocardial infarction and stroke [1]. Its burden is expected to rise with aging populations, increasing prevalence of obesity, cancer, and chronic cardiopulmonary disease [3].

Population-level trends in PE mortality provide important insights into the efficacy of prevention, diagnostic, and treatment strategies. Previous analyses of national databases revealed that mortality decreased in the early 2000s, coinciding with improved preventive and treatment regimens [4]. However, recent findings indicate a plateau, if not a revival, in specific demographic groups, reflecting the increased burden of contributing causes of death, gaps in healthcare access, and the limitations of current preventative strategies [5]. Persistent disparities in sex, race, geography, and socioeconomic position underline the need for a new, data-driven public health focus.

Despite its clinical significance, current national investigations of PE mortality are few, and to our knowledge, none have predicted its future trajectory. Current evidence mostly ends before 2020 and rarely includes predictive modeling. Leveraging the Centers for Disease Control and Prevention’s Wide-ranging Online Data for Epidemiologic Research (CDC WONDER) database offers a unique opportunity to bridge this gap. This study uses national mortality data from 1968–2023 to examine temporal trends in PE-related deaths among US adults aged ≥ 25 years. It employs autoregressive integrated moving average (ARIMA) and Prophet models to forecast future patterns. By utilizing retrospective surveillance with modern predictive analytics, this analysis provides an updated national representation of PE mortality, identifies persistent disparities, and provides data-driven forecasts critical for guiding prevention, health system planning, and policy action to reduce future PE related deaths.

## Methods

### Study setting and population

We conducted a descriptive analysis using death certificate data from the Centers for Disease Control and Prevention's Wide-Ranging Online Data for Epidemiologic Research database [6]. Pulmonary embolism (PE) deaths were identified in individuals ≥ 25 years of age, with International Classification of Diseases-8 (ICD-8-CM) codes 450 for the years 1968 to 1978, ICD-9-CM codes 415.1 for the years 1979 to 1998 and ICD-10-CM codes I26 from 1999 onwards. We applied a consistent definition of PE across ICD revisions. For the period 1999–2023, we further identified the most common contributing causes of death among individuals with PE as the underlying cause, including coronary artery disease (ICD-10-CM codes I20–I25), heart failure (I50), hypertension (I10-I15), diabetes mellitus (E10–E14), chronic obstructive pulmonary disease (J40–J47), and infections comprised of sepsis (A02.1, A20.7, A22.7, A26.7, A32.7, A40.0, A40.1, A40.2, A40.3, A40.8, A40.9, A41.0, A41.1, A41.2, A41.3, A41.4, A41.5, A41.8, A41.9, A42.7, and B37.7) and Pneumonia (J12-J18). Hypertension was included as a contributing cause because it is commonly recorded on death certificates and reflects overall cardiometabolic burden, although it is not consistently established as an independent causal risk factor for PE in prior epidemiologic literature.

Our analysis focused on death certificates from the database, capturing cases where pulmonary embolism was listed as a primary cause of death. Institutional review board approval was not required, as we used a de-identified, publicly available dataset in compliance with Strengthening the Reporting of Observational Studies in Epidemiology (STROBE) guidelines [7].

### Data abstraction

We categorized data by demographic factors, including age, sex, and race. Age groups were categorized as adults aged 25–44 years (younger adults), 45–64 years (middle-aged adults), and 65 + (older adults). Racial groups included White and Black/African American.

### Statistical analysis

To standardize related deaths, age-adjusted mortality rates (AAMRs) per 100,000 population were calculated using the annual national population totals from the US Census Bureau and the 2000 US standard population, along with the corresponding 95% confidence intervals (CIs) [8]. Temporal trends were assessed using the Joinpoint Regression version 5.2.0 (National Cancer Institute) [9]. Annual percentage changes (APCs) with 95% CIs were calculated among intervals identified by the Joinpoint regression. APCs were categorized as increasing or decreasing based on statistical deviation from the null hypothesis of zero change. Statistical significance was determined using a two-tailed t-test with a significance threshold of P < 0.05.

### Time-series forecasting

The forecasting of pulmonary embolism-related deaths through 2040 was performed using a data-driven approach based on CDC WONDER mortality data. Two forecasting models were evaluated: (1) the Autoregressive Integrated Moving Average (Auto-ARIMA) model and (2) the Prophet model. A fixed random seed was applied at each fitting step to ensure reproducibility. All analyses were conducted using R version 4.5.0. We evaluated two established time-series forecasting approaches: automated autoregressive integrated moving average (Auto-ARIMA) and Prophet. Auto-ARIMA is a statistical model that forecasts future values using past observations of the outcome by modeling autocorrelation patterns through autoregressive and moving-average terms after differencing to achieve stationarity. Prophet is an additive regression-based time-series model that decomposes observed values into a long-term trend component, potential changepoints, and an error term; because our dataset consisted of annual mortality rates, seasonal components were not applicable. Both models were implemented as univariate models, using only the annual age-adjusted mortality rate (AAMR) time series within each stratum. These forecasting models were not supplied with external predictors (e.g., projected PE incidence, prevalence of risk factors, or population-level comorbidity projections); therefore, forecasts represent trend-based extrapolations of historical mortality patterns rather than mechanistic predictions driven by future incidence.

Three training windows were applied for demographic projections (sex, age group, and race): 1968–2015, 1988–2015, and 1999–2015. Model performance was validated against observed data from 2016–2023, and forecasts were then extended through 2040. For each stratum, the model with the lowest RMSE was selected as the optimal forecasting approach. For contributing cause of death projections, a single training period (1999–2015) was used, with validation from 2016–2023 and forecasts generated through 2040, again selecting the best-performing model based on RMSE.

After model selection based on validation performance, the selected model for each stratum was refitted using the full observed dataset through 2023, and final forecasts were generated for 2024–2040.

Prophet does not inherently incorporate projected incidence unless external regressors are explicitly provided; because CDC WONDER does not provide incidence data and robust national incidence projections were not available for integration, projected incidence was not included in this analysis.

For contributing cause analyses (1999–2023), we modeled annual AAMRs for each contributing cause category among decedents with PE as the underlying cause of death. Auto-ARIMA and Prophet models were trained using 1999–2015 data and validated against observed 2016–2023 values. RMSE was used to identify the optimal model for each contributing cause time series, and the selected model was then refitted using the full 1999–2023 dataset to generate forecasts through 2040.

### Model validation and selection

Forecasting models were evaluated using out-of-sample validation against observed AAMR values from 2016–2023. For each stratum (overall, sex, age group, and race), the model with the lowest root mean square error (RMSE) was selected as the final forecasting approach. Model performance metrics for Auto-ARIMA and Prophet across training windows are reported in Supplemental Table 6. Overall, both models demonstrated good agreement with observed 2016–2023 trends, with the selected model varying by stratum depending on the historical time window.

## Results

### Annual trends

**Fig. 1:**
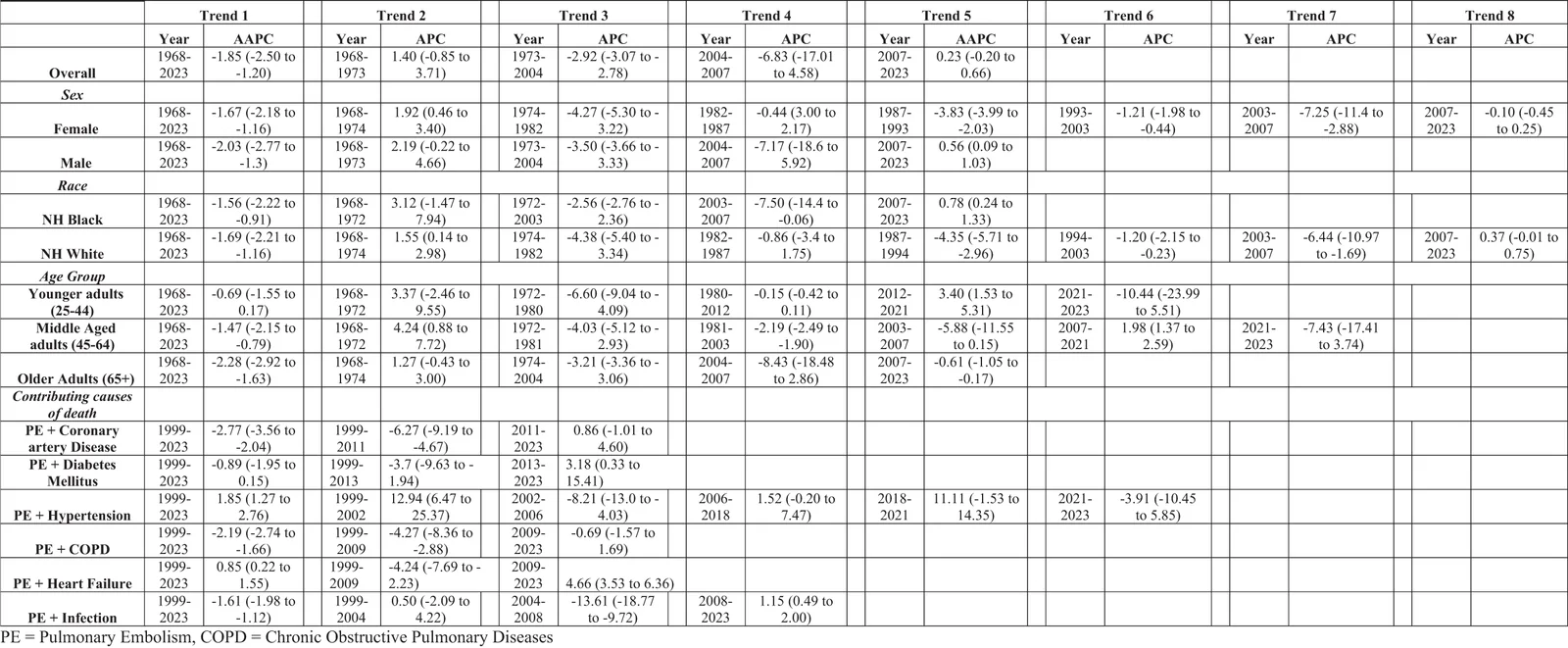
Annual percent change (APC) in pulmonary embolism-related mortality by total population, sex, race, age, and contributing cause of death, 1968–2023

**Fig. 2:**
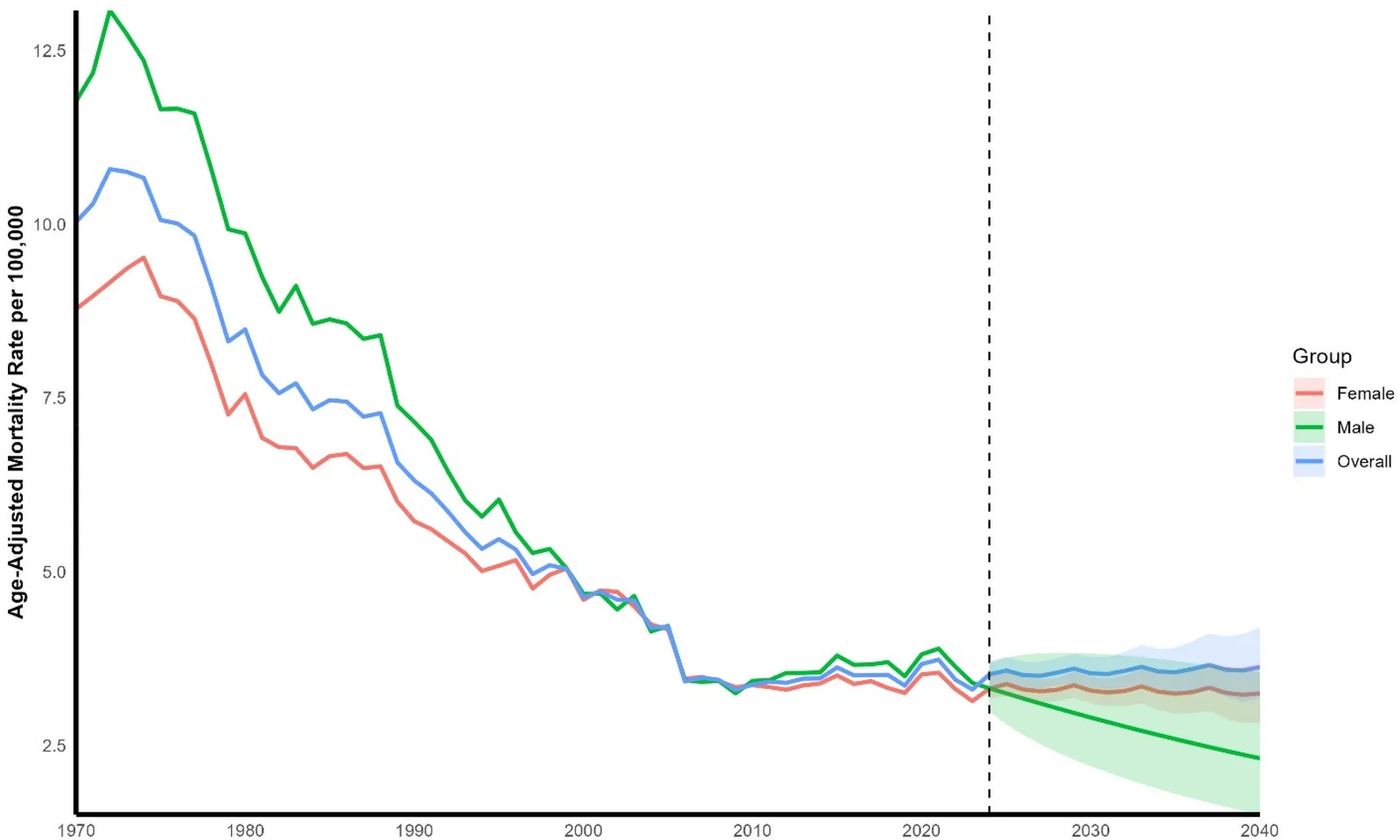
Pulmonary embolism-related AAMR stratified by overall and sex per 100,000 population

Between 1968 and 2023, a total of 516,187 pulmonary embolism-related deaths were recorded (Supplemental Table 1). AAMR dropped from 9.92 (95% CI: 9.71 to 10.1) in 1968 to 3.31 (95% CI: 3.24 to 3.38) in 2023 (AAPC: -1.85%; CI: -2.50 to -1.20). Highest overall AAMR of 10.79 (95% CI: 10.59 to 11.0) existed in 1972 **(**Figs. 1, 2**)** (Supplemental Table 2, 6).

By 2040, the overall AAMR is projected to reach 3.63 (95% CI: 3.18 to 4.20).

### Sex

Males had 239,838 deaths from 1968 to 2023. Males consistently had higher mean AAMRs (mean AAMR: 6.76, 95% CI: 6.56 to 6.97). In 1968, male AAMR was 11.8 (95% CI: 11.4 to 12.1), dropping to 3.40 (95% CI: 3.3 to 3.51) by 2023 with an AAPC of -2.03%, 95% CI: -2.77 to -1.3).

Females reported 276,349 deaths from 1968 to 2023. AAMR among females declined from 8.55 (95% CI: 8.29 to 8.8) in 1968 to 3.14 (95% CI: 3.05 to 3.24) in 2023 (AAPC: -1.67%; CI: -2.18 to -1.16) (Figs. 1, 2) (Supplemental Table 2, 6).

We projected that the male AAMR will reach to 2.32 (95% CI: 1.51 to 3.56) by 2040 and female AAMR at 3.25 (95% CI: 2.83 to 3.69) by 2040.

### Race

**Fig. 3:**
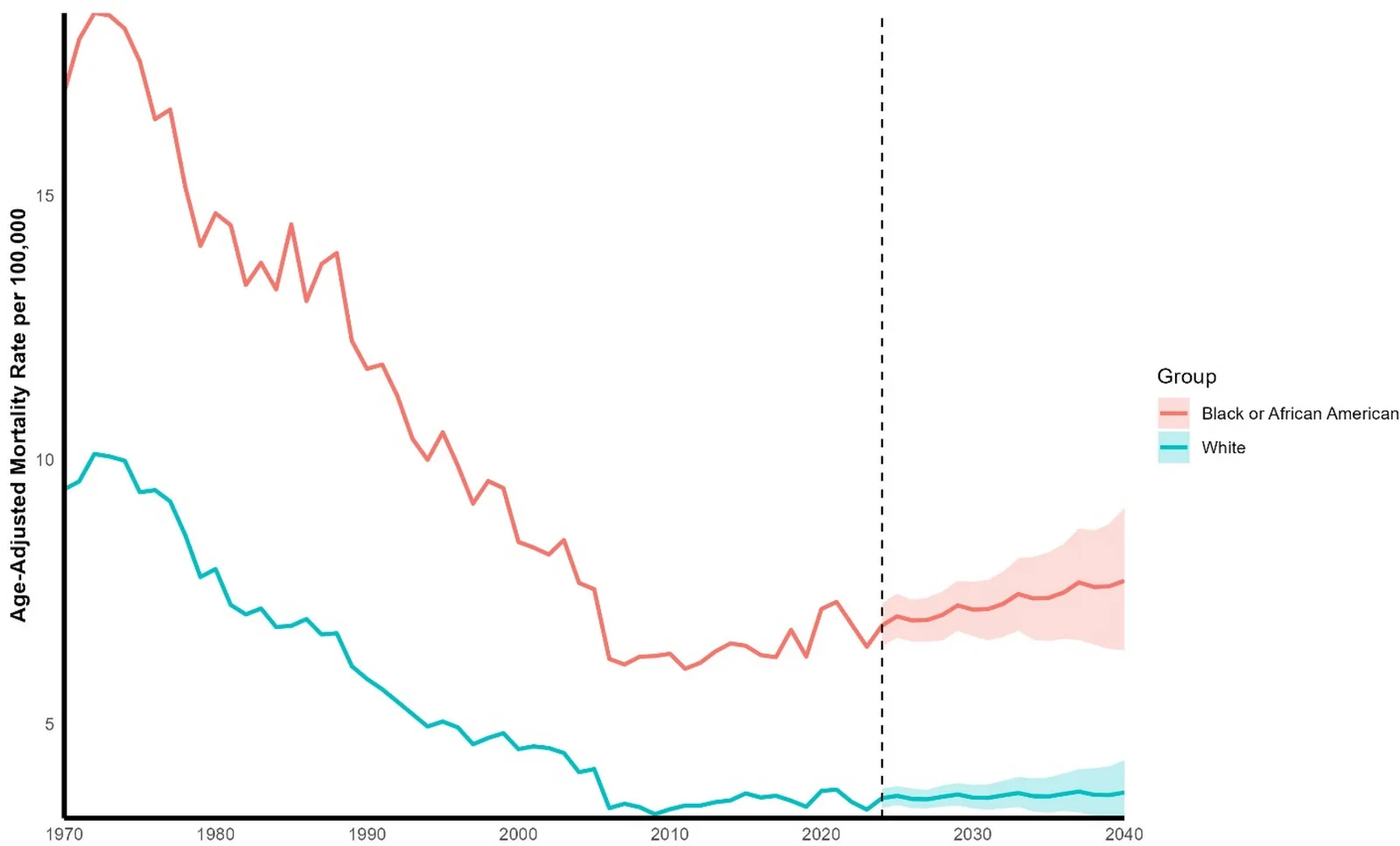
Pulmonary embolism-related AAMR stratified by race per 100,000 population

Between 1968 and 2023, 416,742 pulmonary embolism-related deaths occurred among White adults. AAMR among whites decreased from 9.30 (95% CI: 9.09 to 9.51) in 1968 to 3.38 (95% CI: 3.29 to 3.45) in 2023 (AAPC: -1.69%; CI: -2.21 to -1.16; p value < 0.001). In Black adults, only 86,869 deaths occurred from 1968 to 2023. AAMR decreased from 16.7 (95% CI: 15.8 to 17.6) in 1968 to 6.46 (95% CI: 6.15 to 6.77) in 2023 (AAPC: -1.56%; CI: -2.22 to -0.91; p value = 0.003) (Fig. 3) (Supplemental Table 3,6).

Projections for 2040 estimate White adults AAMR at 3.70 (95% CI: 3.24 to 4.31) and Black adults AAMR at 7.71 (95% CI: 6.39 to 9.09).

### Age

From 1968 to 2023, there were 38,162 deaths among younger adults (25–44 years), 128,581 among middle-aged adults (45–64 years), and 349,444 among older adults (≥ 65 years) (Supplemental Fig. 1) (Supplemental Table 4, 6). When stratified by age, PE mortality declined most in older adults (AAPC: -2.27, 95% CI: -2.92 to -1.63; p value < 0.001), moderately in middle-aged adults; (AAPC: -1.47, 95% CI: -2.14 to -0.78; p value = 0.025), and showed a slight, non-significant change in younger adults (AAPC: -0.69, 95% CI: -1.54 to 0.17; p value = 0.116).

By 2040, AAMR is projected at 1.08 (95% CI: 0.97 to 1.20) for younger adults, 2.81 (95% CI: 2.28 to 3.46) for middle-aged adults, and 9.24 (95% CI: 7.88 to 10.57) for older adults.

### Comorbidities

From 1999 to 2023, the AAMR for coronary artery disease-related pulmonary embolism decreased from 0.48 (95% CI: 0.45 to 0.52) to 0.25 (95% CI: 0.23 to 0.27), with an AAPC of -2.77% (95% Cl: -3.56 to -2.04; p value < 0.001). By 2040, the AAMR is projected to reach 0.25 (95% CI: 0.09 to 0.69).

For diabetes-related pulmonary embolism, the AAMR decreased from 0.28 (95% CI: 0.26 to 0.31) in 1999 to 0.21 (95% Cl: 0.19 to 0.22) in 2023 with an AAPC of -0.89% (95% CI: -1.95 to 0.15; p value = 0.094). The AAMR was projected to 0.21 (95% CI: 0.07 to 0.59) by 2040.

The AAMR for hypertension-related pulmonary embolism increased from 0.23 (95% Cl: 0.21 to 0.25) in 1999 to 0.37 (95% Cl: 0.35 to 0.39) in 2023 with an AAPC of 1.85% (95% CI: 1.27 to 2.76; p value < 0.001). AAMR was projected to 0.37 (95% CI: 0.15 to 0.95) by 2040.

For chronic obstructive disease-related pulmonary embolism, the AAMR decreased from 0.33 (95% CI: 0.30 to 0.36) in 1999 to 0.18 (95% CI: 0.17 to 0.2) in 2023 with an AAPC of -2.19% (95% CI: -2.74 to -1.66; p value < 0.001). AAMR was projected to 0.13 (95% CI: 0.08 to 0.20) by 2040.

For heart failure-related pulmonary embolism, the AAMR stabilized from 0.33 (95% Cl: 0.30 to 0.36) in 1999 to 0.38 (95% Cl: 0.35 to 0.40) in 2023 with an AAPC of 0.85% (95% CI: 0.22 to 1.55; p value = 0.009). AAMR was projected to 0.72 (95% CI: 0.10 to 5.04) by 2040.

For infection-related pulmonary embolism, the AAMR decreased from 0.28 (95% Cl: 0.26 to 0.30) in 1999 to 0.19 (95% Cl: 0.17 to 0.21) in 2023 with an AAPC of -1.61% (95% CI: -1.98 to -1.12; p < 0.001). The AAMR was projected to 0.19 (95% CI: 0.09 to 0.41) by 2040 (Supplemental Fig. 2) (Supplemental Table 5, 7).

## Discussion

**Fig. 4:**
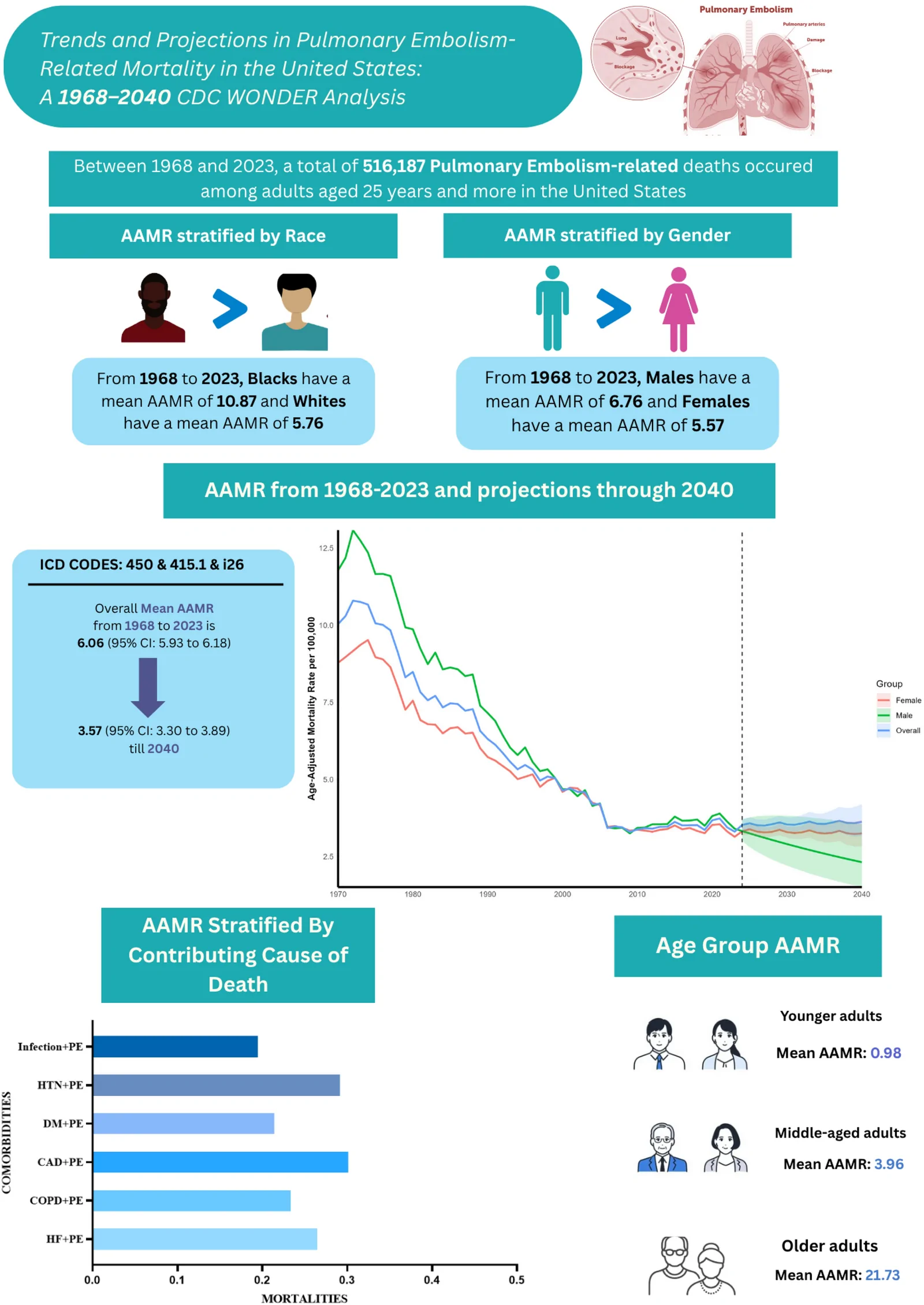
Trends and projections in pulmonary embolism-related mortality in the United States: a 1968–2040 CDC WONDER analysis

Our study provides a comprehensive 55-year analysis of PE trends among individuals aged ≥ 25 in the U.S., with projection through 2040 **(**Fig. 4**)**. Mortality rates decreased throughout the study, with the steepest decline between 2004 and 2007. Men consistently showed higher mortality rates compared to women. Racial and age-related disparities were observed, with NH Black adults and older adults having higher mortality rates compared to NH White adults and other age groups. PE mortality related to contributing causes of death, including coronary artery disease, diabetes, chronic obstructive pulmonary disease, and infections, declined, whereas deaths associated with heart failure and hypertension increased or fluctuated. Overall mortality due to PE is projected to increase by approximately 9.7% by 2040 based on extrapolation of historical mortality trends, although these projections should be interpreted cautiously because future changes in prevention, diagnosis, and treatment may alter these trajectories. These findings underscore the need for targeted public health strategies and interventions to reduce the overall mortality burden.

During the whole study period from 1968 till 2023, mortality rates due to PE showed distinct trends. The mortality rates slightly increased in the early 1970s; this early rise in the mortality rates might be attributed to lack of awareness, lesser diagnostic measures, and fewer effective treatments at that time [10]. From the mid-1970s till the early 2000s, the mortality rates gradually decreased, with the sharpest decline between 2004 and 2007; this reversal in the trend may be presumably due to improvement in anticoagulant therapy, better hospital care, and increased recognition of risk factors at that time [11, 12]. Additionally, the use of computed tomography in patients with PE resulted in better outcomes and treatment, as indicated by Enden and Kløw [13]. More importantly, after 2007, the mortality rates nearly plateaued, suggesting that the early gains in the mortality rates have been counteracted by certain factors, including an increased aging population and increased prevalence of comorbidities such as diabetes, obesity, and hypertension [14–16]. Projections suggest a slight increase in mortality rates through 2040. However, these estimates represent statistical extrapolations of prior trends rather than causal predictions, and future shifts in comorbidity burden, treatment strategies, or population demographics may modify these trajectories [17]. These findings underscore the need for targeted healthcare approaches and public health awareness to reduce the mortality rates.

Males have a higher baseline risk of venous thromboembolism due to hormonal differences, vascular physiology, and coagulation profile [18]. Behavioral factors such as higher prevalence of smoking, higher prevalence of obesity, and lesser health-seeking behavior compound the risk of PE among men [19–21]. Additionally, males are likely to have more comorbidities, including cardiovascular diseases, metabolic disorders, and acute cardiac events [22, 23]. In contrast, women in their early life remain relatively protected because of high estrogen levels before menopause [24]. Interestingly, the projections indicate that the mortality rates among females will surpass the mortality rates among men till 2040. This projected convergence likely reflects demographic aging patterns captured in the model rather than a definitive biological shift, and should therefore be interpreted cautiously. These findings reflect important sex disparities and appeal for sex-specific interventional approaches and vigilant management, especially in older women. In addition, long-range sex-specific forecasts are inherently uncertain, and the predicted divergence between male and female mortality trajectories should be interpreted as a model-based extrapolation rather than a definitive biological shift. Sex differences in VTE and PE risk are complex and vary substantially across the life course. While several studies report higher VTE recurrence risk among men, PE risk in women may be increased during periods of hormonal exposure (e.g., pregnancy, postpartum, and oral contraceptive use), and sex differences narrow or change direction across older age groups. Accordingly, long-term PE mortality patterns likely reflect an interaction between age distribution, comorbidity burden, hormonal factors, and healthcare access rather than a uniform sex-specific biological risk.

Persistent racial disparities were identified, with Black adults consistently showing higher AAMR compared to White adults. While overall mortality rates declined for both groups, the reduction has been more pronounced among White adults, reflecting important differences in access to healthcare, early diagnosis, and effective management strategies. Black individuals historically have a higher prevalence of contributing causes of deaths, including hypertension, diabetes, and obesity, which increase the risk of severe outcomes in PE [25, 26]. Moreover, Black individuals face additional structural barriers such as limited access to medical healthcare facilities, socioeconomic inequalities, and lack of healthcare literacy, which can further contribute to the higher mortality rates among Black adults [27, 28]. compared to White adults, which can be explained by the same above-mentioned reasons. Projections indicated nearly twice the increase in AAMR among Black adults compared with White adults. These projections likely reflect continuation of historical disparities captured in the dataset rather than causal inference, but they highlight populations that may remain vulnerable if current inequities persist. Persistently higher mortality rates among older adults can be explained by several factors, including higher prevalence of chronic conditions and comorbidities such as hypertension, heart failure, diabetes mellitus, and obesity [29], which not only increase the chances of thromboembolism but also complicate treatment [30]. Similarly, middle-aged individuals have shown relatively higher mortality rates compared to younger adults due to occupational sedentary behaviour which can result in worsening outcomes in PE [31]. These findings appeal for large-scale public health strategies and interventional approaches to decrease mortality rates, especially among older individuals.

PE associated with contributing causes of death showed overall heterogeneous trends, reflecting both disease management strategies and underlying risk factors. Firstly, mortality linked to coronary artery disease and chronic obstructive pulmonary disease declined throughout the study, with projections indicating further decreases in the mortality rates, likely due to disease-specific management, widespread use of anticoagulant therapy, and better control of cardiovascular and pulmonary risk factors [32, 33]. PE with diabetes had shown a steady decline before 2013, then a persistent increase in the mortality rates, which may be attributed to higher prevalence of obesity, metabolic syndrome, and poor glycemic control [34], all of which can lead to a hypercoagulable state and subsequent thromboembolism. However, the mortality rates due to PE and DM are predicted to stabilize at the lower side till 2040. This predicted lower AAMR may be explained by improvements across prevention, diagnosis, and treatment. Early detection through routine risk assessment, D-dimer testing, and screening programs enables timely intervention, while advances in novel drugs like SGLT2 inhibitors and GLP-1 receptor agonists have significantly reduced complications and deaths [35, 36]. In parallel, national U.S. data demonstrate increasing hypertension prevalence over recent decades, which may contribute to increasing documentation of hypertension as a contributing cause among decedents with PE. In contrast, with hypertension, the mortality rates increased throughout the study period and are projected to continue increasing through 2040 if historical trends persist, the increasing trend in hypertension-listed PE deaths likely reflects rising population prevalence of hypertension and increasing documentation of cardiometabolic comorbidity on death certificates rather than a direct causal relationship between hypertension and PE. Hypertension may therefore act as a marker of overall vascular risk burden, multimorbidity, and healthcare utilization patterns in patients dying with PE [37]. PE with heart failure initially demonstrated improvement in the mortality rates due to optimized cardiac care and use of anticoagulants [32]. However, the reversal in the trend thereafter might be a result of venous stasis, systemic inflammation, and age-related vulnerability, and are projected to continue increasing through 2040 within the forecasting model [38]. Infection-related PE mortality initially decreased due to better infection control and early recognition, then the mortality increased due to ongoing risk from severe infections and sepsis, which could subsequently lead to PE [39], though projections suggest a relative stabilization by 2040. These trends highlight that while advances in anticoagulation, early diagnosis, and hospital care have reduced mortality in some comorbid groups, persistent or rising mortality in others is driven by increasing comorbidity burden, aging populations, and prothrombotic mechanisms. Minor year-to-year fluctuations, such as sharper declines in coronary artery disease and COPD-related PE in the early 2000s and transient increases in diabetes- and hypertension-related PE, illustrate the dynamic interplay between improved treatment and emerging risk factors, emphasizing the need for targeted, multidisciplinary preventive and therapeutic strategies to mitigate projected risks. Therefore, trends in contributing causes should be interpreted as patterns in comorbidity documentation among PE deaths rather than independent incidence trends of PE attributable to each condition.

Health systems should prioritize early identification of high-risk individuals through routine screening, improved risk stratification, and timely initiation of anticoagulant therapy [40, 41]. Additionally, sex- and race-specific interventions, including education, equitable access to care, and culturally tailored management programs, are essential to reduce persistent disparities. Campaigns like “Stop the Clot, Spread the Word®,” developed by the National Blood Clot Alliance in collaboration with the CDC [42], provide an evidence-based framework for raising awareness about blood clots, their risk factors, symptoms, and preventive strategies.

Forecasting estimates should be interpreted as data-driven extrapolations of historical mortality patterns. These models do not incorporate future changes in PE incidence, anticoagulation practices, diagnostic strategies, healthcare access, or major public health disruptions. As such, projections are intended to inform potential future trajectories under continuation of observed trends rather than provide deterministic predictions.

### Limitations

This study is subjected to several limitations. Firstly, relying on the ICD codes and extracting data from death certificates involves a risk of misclassification or underreporting PE as a cause of death. Secondly, higher use of electronic health records for diagnosing PE may inflate reported cases on death certificates, which could distort perceived trends in pulmonary embolism-related mortality. Thirdly, certain variables, including patients’ socioeconomic status and laboratory findings could not be assessed due to the absence of relevant data. Cancer was not included among contributing causes because cancer-related coding on death certificates is highly heterogeneous across many ICD-10 categories, and grouping malignancies into a single category may introduce misclassification and reduce interpretability. Given the strong association between malignancy and PE, future work using cancer registry-linked datasets would be better suited to evaluate cancer-associated PE mortality. Although sex-specific forecasts suggested divergence in projected AAMR trajectories, historical PE mortality trends for men and women were broadly parallel across the long-term study period. Accordingly, sex-specific projections should be interpreted cautiously, particularly beyond the near-term forecast horizon, given that forecasting models extrapolate recent trend structures and may amplify short-term fluctuations observed during the validation period. Rather than being exact predictions of future occurrences or the results of clinical or policy interventions, our projections should be understood as trend-based estimations. Unexpected events like breakthrough treatments, public health initiatives, or pandemics in the future cannot be taken into account by these models. There are still inherent limitations, such as the possibility of under- or overestimation, even though the model was chosen based on objective performance metrics, especially RMSE, and validated using out-of-sample data to minimize overfitting. Crucially, in order to preserve transparency on the level of confidence that the data and model support, we did not purposefully suppress or narrow these intervals.

## Conclusion

The long-term analysis of mortality rates due to PE in the U.S. has shown an overall decline in mortality trends, with persistent disparities across sex, race, age, and contributing causes of death, with Black adult and older women having higher mortality rates throughout the study period, with projections indicating higher mortality rates among women by 2040. Contributing causes of death, especially hypertension and heart failure, have been observed to increase the risk of PE-related mortality and are projected to further increase mortality rates by 2040. These findings underscore the need for targeted, multidimensional approaches and healthcare strategies to reduce the burden of mortality.

## Supplementary Information

Below is the link to the electronic supplementary material.

Supplementary file1 (DOCX 256 kb)

## Data Availability

The data supporting the findings of this study were obtained from the CDC WONDER online database (Centers for The data supporting the findings of this study were obtained from the CDC WONDER online database (Centers for Disease Control and Prevention Wide-ranging Online Data for Epidemiologic Research). The datasets used and analyzed during the current study are publicly available and can be accessed at [CDC WONDER] (https:/wonder.cdc.gov).
