# Supplementary material for: Temporal Trends and Future Projections of P ulmonary Embolism and Top Contributing Causes of death in the United States, 1968–2040: insights from the CDC WONDER database
Source: J Thromb Thrombolysis. 2026 May 24;59(7):1985–95. doi: 10.1007/s11239-026-03281-9 (PMC13654879; doi:10.1007/s11239-026-03281-9)
Supplement: Supplementary file 1 [file 11239_2026_3281_MOESM1_ESM.docx]

**Supplemental Table 1, Pulmonary Embolism related mortalities in Adults, Stratified by Sex and Race, in the United States, 1968 to 2023**

| **Supplemental Table 1, Pulmonary Embolism related mortalities in Adults, Stratified by Sex and Race, in the United States, 1968 to 2023** | | | | | | |
| --- | --- | --- | --- | --- | --- | --- |
| **Deaths** | | | | | | |
| **Year** | **Overall** | **Women** | **Men** | **Whites** | **Black or African American** | **Population** |
| **1968** | 9562 | 4604 | 4958 | 8084 | 1436 | 107469000 |
| **1969** | 9764 | 4781 | 4983 | 8243 | 1488 | 108734000 |
| **1970** | 10171 | 5011 | 5160 | 8621 | 1514 | 109870505 |
| **1971** | 10630 | 5230 | 5400 | 8921 | 1663 | 111675000 |
| **1972** | 11446 | 5492 | 5954 | 9648 | 1760 | 114147000 |
| **1973** | 11513 | 5738 | 5775 | 9704 | 1756 | 116274000 |
| **1974** | 11591 | 5945 | 5646 | 9769 | 1773 | 118403000 |
| **1975** | 11246 | 5787 | 5459 | 9447 | 1751 | 120564000 |
| **1976** | 11391 | 5883 | 5508 | 9649 | 1691 | 122914000 |
| **1977** | 11492 | 5867 | 5625 | 9676 | 1756 | 125376000 |
| **1978** | 10835 | 5542 | 5293 | 9150 | 1646 | 127957000 |
| **1979** | 10087 | 5168 | 4919 | 8479 | 1551 | 130668000 |
| **1980** | 10470 | 5482 | 4988 | 8794 | 1618 | 132768638 |
| **1981** | 9899 | 5169 | 4730 | 8245 | 1610 | 136009066 |
| **1982** | 9794 | 5196 | 4598 | 8219 | 1541 | 138690289 |
| **1983** | 10192 | 5303 | 4889 | 8527 | 1605 | 141303090 |
| **1984** | 9880 | 5194 | 4686 | 8244 | 1592 | 143881783 |
| **1985** | 10241 | 5408 | 4833 | 8430 | 1762 | 146398041 |
| **1986** | 10418 | 5552 | 4866 | 8756 | 1606 | 149041120 |
| **1987** | 10282 | 5495 | 4787 | 8519 | 1719 | 151539755 |
| **1988** | 10534 | 5598 | 4936 | 8680 | 1790 | 153897145 |
| **1989** | 9629 | 5250 | 4379 | 7978 | 1591 | 156206784 |
| **1990** | 9376 | 5067 | 4309 | 7762 | 1542 | 157843690 |
| **1991** | 9343 | 5094 | 4249 | 7693 | 1602 | 161231105 |
| **1992** | 9144 | 5039 | 4105 | 7540 | 1549 | 163875074 |
| **1993** | 8865 | 4955 | 3910 | 7333 | 1485 | 166358177 |
| **1994** | 8628 | 4790 | 3838 | 7108 | 1467 | 168791121 |
| **1995** | 9007 | 4930 | 4077 | 7354 | 1583 | 171332456 |
| **1996** | 8934 | 5117 | 3817 | 7338 | 1519 | 173893568 |
| **1997** | 8494 | 4795 | 3699 | 6978 | 1445 | 176251758 |
| **1998** | 8863 | 5079 | 3784 | 7273 | 1519 | 178366837 |
| **1999** | 8905 | 5220 | 3685 | 7054 | 1509 | 180408769 |
| **2000** | 8339 | 4837 | 3502 | 6677 | 1367 | 181984640 |
| **2001** | 8551 | 5010 | 3541 | 6830 | 1388 | 184305128 |
| **2002** | 8536 | 5054 | 3482 | 6798 | 1386 | 186208028 |
| **2003** | 8602 | 4907 | 3695 | 6794 | 1469 | 188090429 |
| **2004** | 8027 | 4679 | 3348 | 6323 | 1346 | 190205384 |
| **2005** | 8192 | 4687 | 3505 | 6480 | 1358 | 192551384 |
| **2006** | 6856 | 3937 | 2919 | 5374 | 1162 | 195019359 |
| **2007** | 7019 | 4038 | 2981 | 5553 | 1152 | 197403777 |
| **2008** | 7079 | 4018 | 3061 | 5538 | 1209 | 199795090 |
| **2009** | 6956 | 3945 | 3011 | 5341 | 1270 | 202107016 |
| **2010** | 7233 | 4033 | 3200 | 5609 | 1289 | 203891983 |
| **2011** | 7440 | 4112 | 3328 | 5762 | 1302 | 206592936 |
| **2012** | 7600 | 4113 | 3487 | 5864 | 1345 | 208826037 |
| **2013** | 7829 | 4246 | 3583 | 6042 | 1412 | 211085314 |
| **2014** | 8072 | 4380 | 3692 | 6145 | 1501 | 213809280 |
| **2015** | 8578 | 4568 | 4010 | 6553 | 1552 | 216553817 |
| **2016** | 8403 | 4450 | 3953 | 6370 | 1534 | 218641417 |
| **2017** | 8624 | 4569 | 4055 | 6529 | 1563 | 221447331 |
| **2018** | 8731 | 4566 | 4165 | 6443 | 1719 | 223311190 |
| **2019** | 8527 | 4487 | 4040 | 6338 | 1648 | 224981167 |
| **2020** | 9286 | 4853 | 4433 | 6711 | 1931 | 226635013 |
| **2021** | 9362 | 4810 | 4552 | 6706 | 1926 | 228238412 |
| **2022** | 9032 | 4719 | 4313 | 6486 | 1830 | 229508599 |
| **2023** | 8687 | 4550 | 4137 | 6260 | 1771 | 231529762 |
| **Total** | **516187** | **276349** | **239838** | **416742** | **86869** | **9544862264** |

**Supplemental Table 2 Overall and Sex‐Stratified Pulmonary Embolism related Age-Adjusted Mortality Rates per 100,000, in Adults in the United States, 1968 to 2023**

| **Supplemental Table 2 Overall and Sex‐Stratified Pulmonary Embolism related Age-Adjusted Mortality Rates per 100,000, in Adults in the United States, 1968 to 2023** | | | |
| --- | --- | --- | --- |
| **Age-Adjusted Rate (95% CI)** | | | |
| **Year** | **Men** | **Women** | **Overall** |
| **1968** | 11.77 (11.414 - 12.126) | 8.55 (8.295 - 8.805) | 9.918 (9.71 - 10.127) |
| **1969** | 11.746 (11.393 - 12.1) | 8.683 (8.43 - 8.936) | 9.962 (9.755 - 10.169) |
| **1970** | 11.777 (11.436 - 12.118) | 8.781 (8.533 - 9.029) | 10.035 (9.834 - 10.236) |
| **1971** | 12.182 (11.835 - 12.53) | 8.969 (8.721 - 9.217) | 10.297 (10.094 - 10.5) |
| **1972** | 13.082 (12.727 - 13.437) | 9.165 (8.918 - 9.411) | 10.796 (10.592 - 11.001) |
| **1973** | 12.74 (12.389 - 13.091) | 9.364 (9.118 - 9.611) | 10.755 (10.553 - 10.958) |
| **1974** | 12.358 (12.014 - 12.702) | 9.521 (9.275 - 9.767) | 10.67 (10.47 - 10.87) |
| **1975** | 11.659 (11.33 - 11.988) | 8.965 (8.731 - 9.199) | 10.063 (9.872 - 10.254) |
| **1976** | 11.665 (11.338 - 11.993) | 8.895 (8.665 - 9.126) | 10.012 (9.824 - 10.201) |
| **1977** | 11.597 (11.275 - 11.919) | 8.642 (8.418 - 8.866) | 9.837 (9.653 - 10.022) |
| **1978** | 10.791 (10.482 - 11.099) | 7.987 (7.774 - 8.2) | 9.114 (8.939 - 9.29) |
| **1979** | 9.93 (9.636 - 10.224) | 7.262 (7.061 - 7.462) | 8.314 (8.148 - 8.479) |
| **1980** | 9.87 (9.58 - 10.16) | 7.556 (7.354 - 7.759) | 8.487 (8.321 - 8.653) |
| **1981** | 9.244 (8.965 - 9.522) | 6.926 (6.735 - 7.117) | 7.834 (7.676 - 7.991) |
| **1982** | 8.74 (8.473 - 9.007) | 6.792 (6.605 - 6.979) | 7.568 (7.415 - 7.72) |
| **1983** | 9.115 (8.846 - 9.384) | 6.777 (6.593 - 6.962) | 7.712 (7.56 - 7.865) |
| **1984** | 8.568 (8.31 - 8.827) | 6.495 (6.317 - 6.673) | 7.337 (7.191 - 7.484) |
| **1985** | 8.632 (8.376 - 8.887) | 6.663 (6.483 - 6.842) | 7.469 (7.322 - 7.615) |
| **1986** | 8.573 (8.32 - 8.825) | 6.694 (6.516 - 6.872) | 7.446 (7.301 - 7.591) |
| **1987** | 8.351 (8.104 - 8.599) | 6.49 (6.317 - 6.664) | 7.23 (7.089 - 7.372) |
| **1988** | 8.404 (8.159 - 8.649) | 6.517 (6.345 - 6.689) | 7.281 (7.14 - 7.421) |
| **1989** | 7.391 (7.163 - 7.62) | 6.011 (5.847 - 6.175) | 6.573 (6.44 - 6.705) |
| **1990** | 7.154 (6.932 - 7.377) | 5.727 (5.568 - 5.886) | 6.313 (6.184 - 6.442) |
| **1991** | 6.9 (6.685 - 7.116) | 5.614 (5.459 - 5.77) | 6.129 (6.004 - 6.254) |
| **1992** | 6.438 (6.234 - 6.642) | 5.438 (5.287 - 5.589) | 5.864 (5.743 - 5.985) |
| **1993** | 6.031 (5.835 - 6.226) | 5.267 (5.119 - 5.414) | 5.573 (5.457 - 5.69) |
| **1994** | 5.793 (5.604 - 5.982) | 5.012 (4.869 - 5.155) | 5.33 (5.217 - 5.443) |
| **1995** | 6.039 (5.848 - 6.23) | 5.087 (4.944 - 5.23) | 5.47 (5.356 - 5.583) |
| **1996** | 5.57 (5.388 - 5.751) | 5.168 (5.025 - 5.31) | 5.321 (5.211 - 5.432) |
| **1997** | 5.27 (5.096 - 5.445) | 4.76 (4.624 - 4.895) | 4.967 (4.861 - 5.072) |
| **1998** | 5.329 (5.155 - 5.502) | 4.956 (4.819 - 5.093) | 5.095 (4.988 - 5.201) |
| **1999** | 5.05 (4.883 - 5.216) | 5.049 (4.911 - 5.186) | 5.043 (4.938 - 5.148) |
| **2000** | 4.685 (4.527 - 4.843) | 4.599 (4.469 - 4.73) | 4.64 (4.54 - 4.739) |
| **2001** | 4.688 (4.531 - 4.845) | 4.734 (4.602 - 4.865) | 4.713 (4.613 - 4.813) |
| **2002** | 4.459 (4.308 - 4.61) | 4.712 (4.582 - 4.843) | 4.6 (4.503 - 4.698) |
| **2003** | 4.653 (4.5 - 4.806) | 4.504 (4.378 - 4.631) | 4.588 (4.491 - 4.685) |
| **2004** | 4.143 (4 - 4.286) | 4.243 (4.121 - 4.366) | 4.195 (4.103 - 4.287) |
| **2005** | 4.223 (4.08 - 4.365) | 4.173 (4.052 - 4.293) | 4.209 (4.118 - 4.3) |
| **2006** | 3.441 (3.314 - 3.568) | 3.462 (3.352 - 3.571) | 3.425 (3.343 - 3.506) |
| **2007** | 3.418 (3.293 - 3.543) | 3.49 (3.381 - 3.598) | 3.478 (3.396 - 3.56) |
| **2008** | 3.433 (3.309 - 3.556) | 3.431 (3.324 - 3.539) | 3.447 (3.366 - 3.527) |
| **2009** | 3.253 (3.134 - 3.371) | 3.342 (3.236 - 3.448) | 3.303 (3.225 - 3.381) |
| **2010** | 3.433 (3.312 - 3.554) | 3.37 (3.264 - 3.476) | 3.38 (3.301 - 3.458) |
| **2011** | 3.442 (3.323 - 3.561) | 3.343 (3.239 - 3.447) | 3.42 (3.341 - 3.498) |
| **2012** | 3.547 (3.427 - 3.667) | 3.306 (3.203 - 3.409) | 3.405 (3.327 - 3.482) |
| **2013** | 3.548 (3.429 - 3.666) | 3.367 (3.264 - 3.471) | 3.465 (3.387 - 3.543) |
| **2014** | 3.554 (3.437 - 3.67) | 3.395 (3.292 - 3.498) | 3.468 (3.392 - 3.545) |
| **2015** | 3.796 (3.676 - 3.916) | 3.511 (3.406 - 3.616) | 3.626 (3.548 - 3.704) |
| **2016** | 3.663 (3.547 - 3.78) | 3.388 (3.285 - 3.49) | 3.511 (3.434 - 3.587) |
| **2017** | 3.669 (3.554 - 3.785) | 3.428 (3.325 - 3.53) | 3.516 (3.44 - 3.591) |
| **2018** | 3.701 (3.586 - 3.816) | 3.331 (3.232 - 3.431) | 3.519 (3.444 - 3.595) |
| **2019** | 3.499 (3.389 - 3.609) | 3.259 (3.161 - 3.358) | 3.364 (3.291 - 3.437) |
| **2020** | 3.814 (3.699 - 3.929) | 3.525 (3.423 - 3.628) | 3.675 (3.599 - 3.752) |
| **2021** | 3.895 (3.779 - 4.012) | 3.55 (3.447 - 3.653) | 3.737 (3.659 - 3.814) |
| **2022** | 3.634 (3.522 - 3.745) | 3.314 (3.216 - 3.412) | 3.449 (3.376 - 3.521) |
| **2023** | 3.403 (3.297 - 3.51) | 3.142 (3.048 - 3.236) | 3.308 (3.237 - 3.379) |
| **Mean** | **6.76 (6.55 - 6.96)** | **5.56 (5.41 - 5.72)** | **6.05 (5.93 - 6.18)** |

**Supplemental Table 3 Race‐Stratified Pulmonary Embolism related Age-Adjusted Mortality Rates per 100,000 in Adults in the United States, 1968 to 2023**

| **Supplemental Table 3 Race‐Stratified Pulmonary Embolism related Age-Adjusted Mortality Rates per 100,000 in Adults in the United States, 1968 to 2023** | | |
| --- | --- | --- |
| **Age-Adjusted Rate (95% CI)** | | |
| **Year** | **Black or African American** | **White** |
| **1968** | 16.707 (15.781 - 17.633) | 9.301 (9.088 - 9.513) |
| **1969** | 16.96 (16.046 - 17.875) | 9.342 (9.131 - 9.553) |
| **1970** | 16.954 (16.056 - 17.852) | 9.436 (9.23 - 9.641) |
| **1971** | 17.964 (17.057 - 18.872) | 9.589 (9.383 - 9.794) |
| **1972** | 18.462 (17.561 - 19.364) | 10.107 (9.899 - 10.315) |
| **1973** | 18.415 (17.512 - 19.318) | 10.059 (9.853 - 10.266) |
| **1974** | 18.165 (17.28 - 19.049) | 9.98 (9.777 - 10.184) |
| **1975** | 17.542 (16.685 - 18.398) | 9.383 (9.189 - 9.577) |
| **1976** | 16.45 (15.637 - 17.263) | 9.424 (9.231 - 9.616) |
| **1977** | 16.631 (15.824 - 17.438) | 9.21 (9.022 - 9.397) |
| **1978** | 15.169 (14.409 - 15.929) | 8.572 (8.393 - 8.751) |
| **1979** | 14.05 (13.326 - 14.774) | 7.781 (7.612 - 7.95) |
| **1980** | 14.667 (13.926 - 15.409) | 7.93 (7.761 - 8.099) |
| **1981** | 14.441 (13.709 - 15.172) | 7.252 (7.093 - 7.411) |
| **1982** | 13.308 (12.622 - 13.994) | 7.073 (6.918 - 7.229) |
| **1983** | 13.728 (13.036 - 14.419) | 7.184 (7.029 - 7.338) |
| **1984** | 13.224 (12.556 - 13.891) | 6.835 (6.686 - 6.985) |
| **1985** | 14.457 (13.764 - 15.15) | 6.857 (6.708 - 7.005) |
| **1986** | 13.003 (12.35 - 13.656) | 6.984 (6.836 - 7.132) |
| **1987** | 13.707 (13.044 - 14.371) | 6.693 (6.549 - 6.837) |
| **1988** | 13.913 (13.254 - 14.573) | 6.716 (6.573 - 6.858) |
| **1989** | 12.248 (11.632 - 12.863) | 6.093 (5.958 - 6.228) |
| **1990** | 11.719 (11.12 - 12.317) | 5.852 (5.721 - 5.984) |
| **1991** | 11.801 (11.211 - 12.392) | 5.658 (5.53 - 5.785) |
| **1992** | 11.207 (10.636 - 11.777) | 5.424 (5.301 - 5.547) |
| **1993** | 10.395 (9.856 - 10.935) | 5.188 (5.069 - 5.308) |
| **1994** | 9.996 (9.473 - 10.518) | 4.952 (4.836 - 5.067) |
| **1995** | 10.517 (9.988 - 11.045) | 5.047 (4.931 - 5.163) |
| **1996** | 9.884 (9.377 - 10.392) | 4.935 (4.822 - 5.048) |
| **1997** | 9.17 (8.688 - 9.653) | 4.617 (4.509 - 4.726) |
| **1998** | 9.595 (9.103 - 10.087) | 4.736 (4.627 - 4.845) |
| **1999** | 9.462 (8.975 - 9.948) | 4.826 (4.713 - 4.939) |
| **2000** | 8.443 (7.987 - 8.9) | 4.526 (4.418 - 4.635) |
| **2001** | 8.337 (7.889 - 8.785) | 4.576 (4.467 - 4.685) |
| **2002** | 8.205 (7.763 - 8.647) | 4.545 (4.437 - 4.654) |
| **2003** | 8.479 (8.035 - 8.923) | 4.451 (4.344 - 4.557) |
| **2004** | 7.668 (7.248 - 8.087) | 4.089 (3.988 - 4.19) |
| **2005** | 7.548 (7.136 - 7.96) | 4.148 (4.047 - 4.25) |
| **2006** | 6.231 (5.863 - 6.599) | 3.409 (3.317 - 3.501) |
| **2007** | 6.125 (5.762 - 6.489) | 3.492 (3.399 - 3.585) |
| **2008** | 6.273 (5.91 - 6.637) | 3.427 (3.336 - 3.518) |
| **2009** | 6.286 (5.93 - 6.642) | 3.294 (3.205 - 3.384) |
| **2010** | 6.328 (5.973 - 6.684) | 3.388 (3.297 - 3.478) |
| **2011** | 6.046 (5.708 - 6.384) | 3.457 (3.366 - 3.549) |
| **2012** | 6.157 (5.818 - 6.495) | 3.457 (3.367 - 3.548) |
| **2013** | 6.372 (6.03 - 6.714) | 3.522 (3.431 - 3.614) |
| **2014** | 6.525 (6.186 - 6.864) | 3.556 (3.464 - 3.648) |
| **2015** | 6.479 (6.148 - 6.811) | 3.688 (3.596 - 3.78) |
| **2016** | 6.303 (5.979 - 6.627) | 3.61 (3.518 - 3.702) |
| **2017** | 6.263 (5.944 - 6.581) | 3.642 (3.55 - 3.734) |
| **2018** | 6.781 (6.452 - 7.109) | 3.549 (3.458 - 3.639) |
| **2019** | 6.276 (5.965 - 6.586) | 3.434 (3.346 - 3.532) |
| **2020** | 7.174 (6.846 - 7.502) | 3.733 (3.639 - 3.827) |
| **2021** | 7.309 (6.974 - 7.645) | 3.76 (3.666 - 3.855) |
| **2022** | 6.885 (6.561 - 7.208) | 3.526 (3.436 - 3.616) |
| **2023** | 6.461 (6.152 - 6.769) | 3.381 (3.293 - 3.469) |
| **Mean** | **10.87 (10.31 - 11.42)** | **5.76 (5.63 - 5.89)** |
| NH = Non-Hispanic | | |

**Supplemental Table 4 Age‐Stratified Pulmonary Embolism related Age-Adjusted Mortality Rates per 100,000, in Adults in the United States, 1968 to 2023**

| **Supplemental Table 4 Age‐Stratified Pulmonary Embolism related Age-Adjusted Mortality Rates per 100,000, in Adults in the United States, 1968 to 2023** | | | |
| --- | --- | --- | --- |
| **Age-Adjusted Rate (95% CI)** | | | |
| **Year** | **Younger Adults (25-44)** | **Middle-Aged Adults (45-64)** | **Older Adults (65+)** |
| **1968** | 1.35 (1.244 - 1.457) | 6.085 (5.85 - 6.321) | 36.869 (35.916 - 37.821) |
| **1969** | 1.402 (1.293 - 1.511) | 6.182 (5.947 - 6.418) | 36.799 (35.861 - 37.738) |
| **1970** | 1.35 (1.242 - 1.457) | 6.403 (6.165 - 6.641) | 36.908 (36.002 - 37.815) |
| **1971** | 1.523 (1.409 - 1.638) | 6.556 (6.317 - 6.795) | 37.572 (36.663 - 38.481) |
| **1972** | 1.484 (1.371 - 1.596) | 7.013 (6.767 - 7.259) | 39.418 (38.504 - 40.331) |
| **1973** | 1.419 (1.31 - 1.528) | 6.788 (6.547 - 7.029) | 39.755 (38.843 - 40.668) |
| **1974** | 1.337 (1.231 - 1.443) | 6.532 (6.297 - 6.767) | 39.961 (39.058 - 40.863) |
| **1975** | 1.246 (1.145 - 1.348) | 6.232 (6.003 - 6.461) | 37.597 (36.74 - 38.454) |
| **1976** | 1.171 (1.074 - 1.269) | 5.971 (5.748 - 6.194) | 37.972 (37.122 - 38.823) |
| **1977** | 1.148 (1.053 - 1.243) | 6.075 (5.85 - 6.3) | 36.95 (36.124 - 37.776) |
| **1978** | 1.072 (0.981 - 1.163) | 5.672 (5.455 - 5.889) | 34.139 (33.355 - 34.923) |
| **1979** | 0.934 (0.85 - 1.017) | 5.035 (4.831 - 5.24) | 31.486 (30.744 - 32.228) |
| **1980** | 0.857 (0.777 - 0.936) | 5.052 (4.848 - 5.256) | 32.524 (31.778 - 33.271) |
| **1981** | 0.904 (0.825 - 0.984) | 4.621 (4.425 - 4.816) | 29.829 (29.125 - 30.532) |
| **1982** | 0.912 (0.834 - 0.99) | 4.449 (4.257 - 4.641) | 28.75 (28.071 - 29.43) |
| **1983** | 0.924 (0.846 - 1.002) | 4.697 (4.501 - 4.894) | 29.027 (28.353 - 29.701) |
| **1984** | 0.91 (0.835 - 0.985) | 4.43 (4.24 - 4.62) | 27.612 (26.962 - 28.261) |
| **1985** | 0.882 (0.81 - 0.955) | 4.653 (4.458 - 4.849) | 27.955 (27.309 - 28.6) |
| **1986** | 0.885 (0.813 - 0.956) | 4.531 (4.338 - 4.723) | 28.05 (27.412 - 28.687) |
| **1987** | 0.827 (0.759 - 0.895) | 4.444 (4.253 - 4.635) | 27.232 (26.611 - 27.853) |
| **1988** | 0.852 (0.784 - 0.92) | 4.591 (4.399 - 4.782) | 27.174 (26.56 - 27.789) |
| **1989** | 0.803 (0.738 - 0.869) | 4.036 (3.856 - 4.216) | 24.641 (24.062 - 25.221) |
| **1990** | 0.8 (0.735 - 0.864) | 3.913 (3.737 - 4.09) | 23.537 (22.975 - 24.099) |
| **1991** | 0.809 (0.745 - 0.873) | 3.788 (3.615 - 3.961) | 22.793 (22.25 - 23.337) |
| **1992** | 0.8 (0.737 - 0.862) | 3.723 (3.554 - 3.893) | 21.576 (21.054 - 22.098) |
| **1993** | 0.894 (0.828 - 0.96) | 3.635 (3.469 - 3.8) | 20.019 (19.522 - 20.516) |
| **1994** | 0.894 (0.829 - 0.96) | 3.53 (3.369 - 3.692) | 18.959 (18.48 - 19.438) |
| **1995** | 0.945 (0.879 - 1.012) | 3.694 (3.531 - 3.858) | 19.265 (18.786 - 19.743) |
| **1996** | 0.809 (0.747 - 0.87) | 3.425 (3.27 - 3.58) | 19.299 (18.825 - 19.773) |
| **1997** | 0.801 (0.741 - 0.862) | 3.194 (3.046 - 3.341) | 17.911 (17.458 - 18.363) |
| **1998** | 0.827 (0.765 - 0.888) | 3.201 (3.056 - 3.346) | 18.491 (18.034 - 18.948) |
| **1999** | 0.927 (0.861 - 0.993) | 3.243 (3.099 - 3.387) | 17.917 (17.471 - 18.364) |
| **2000** | 0.882 (0.818 - 0.945) | 3.164 (3.025 - 3.304) | 16.099 (15.678 - 16.52) |
| **2001** | 0.927 (0.861 - 0.993) | 3.243 (3.103 - 3.382) | 16.229 (15.808 - 16.649) |
| **2002** | 0.882 (0.818 - 0.946) | 3.104 (2.971 - 3.237) | 16.004 (15.589 - 16.42) |
| **2003** | 0.982 (0.913 - 1.05) | 3.046 (2.916 - 3.176) | 15.805 (15.395 - 16.216) |
| **2004** | 0.827 (0.766 - 0.888) | 2.75 (2.628 - 2.872) | 14.68 (14.287 - 15.073) |
| **2005** | 0.873 (0.809 - 0.937) | 2.768 (2.648 - 2.888) | 14.613 (14.224 - 15.003) |
| **2006** | 0.773 (0.714 - 0.832) | 2.35 (2.242 - 2.458) | 11.571 (11.227 - 11.915) |
| **2007** | 0.773 (0.713 - 0.832) | 2.311 (2.203 - 2.418) | 11.912 (11.567 - 12.258) |
| **2008** | 0.827 (0.764 - 0.891) | 2.489 (2.38 - 2.599) | 11.309 (10.976 - 11.643) |
| **2009** | 0.827 (0.765 - 0.89) | 2.511 (2.402 - 2.619) | 10.538 (10.218 - 10.857) |
| **2010** | 0.827 (0.765 - 0.889) | 2.511 (2.403 - 2.619) | 10.929 (10.606 - 11.251) |
| **2011** | 0.882 (0.815 - 0.948) | 2.55 (2.442 - 2.658) | 10.935 (10.617 - 11.254) |
| **2012** | 0.882 (0.817 - 0.947) | 2.45 (2.345 - 2.555) | 11.034 (10.718 - 11.35) |
| **2013** | 0.882 (0.816 - 0.948) | 2.611 (2.501 - 2.721) | 11.059 (10.747 - 11.372) |
| **2014** | 0.882 (0.817 - 0.947) | 2.611 (2.503 - 2.719) | 11.078 (10.77 - 11.387) |
| **2015** | 0.936 (0.868 - 1.004) | 2.829 (2.716 - 2.941) | 11.373 (11.064 - 11.682) |
| **2016** | 0.982 (0.913 - 1.051) | 2.85 (2.736 - 2.964) | 10.639 (10.345 - 10.933) |
| **2017** | 0.982 (0.914 - 1.05) | 2.789 (2.678 - 2.901) | 10.771 (10.479 - 11.064) |
| **2018** | 1.036 (0.965 - 1.108) | 2.75 (2.639 - 2.861) | 10.729 (10.442 - 11.016) |
| **2019** | 0.882 (0.818 - 0.946) | 2.871 (2.756 - 2.987) | 10.087 (9.812 - 10.361) |
| **2020** | 1.191 (1.116 - 1.265) | 3.293 (3.168 - 3.418) | 10.209 (9.936 - 10.481) |
| **2021** | 1.191 (1.116 - 1.265) | 3.071 (2.952 - 3.191) | 10.912 (10.625 - 11.198) |
| **2022** | 0.991 (0.924 - 1.058) | 2.789 (2.676 - 2.903) | 10.407 (10.136 - 10.678) |
| **2023** | 0.927 (0.862 - 0.992) | 2.671 (2.559 - 2.784) | 10.043 (9.778 - 10.308) |
| **Mean** | **0.97 (0.90 - 1.05)** | **3.96 (3.79 - 4.12)** | **21.73 (21.19 - 22.26)** |

**Supplemental Table 5 Pulmonary Embolism related Age-Adjusted Mortality Rates per 100,000, Stratified by Comorbidities, in Adults in the United States, 1999 to 2023**

| **Supplemental Table 5 Pulmonary Embolism related Age-Adjusted Mortality Rates per 100,000, Stratified by Comorbidities, in Adults in the United States, 1999 to 2023** | | | | | | |
| --- | --- | --- | --- | --- | --- | --- |
| **Age-Adjusted Rate (95% CI)** | | | | | | |
| **Year** | **PE + coronary artery disease** | **PE + Diabetes Mellitus** | **PE + Hypertension** | **PE + COPD** | **PE + Heart Failure** | **PE + Infection** |
| **1999** | 0.483 (0.451 - 0.515) | 0.284 (0.259 - 0.309) | 0.231 (0.208 - 0.254) | 0.328 (0.302 - 0.355) | 0.33 (0.303 - 0.358) | 0.28 (0.255 - 0.304) |
| **2000** | 0.441 (0.41 - 0.473) | 0.289 (0.263 - 0.314) | 0.286 (0.26 - 0.311) | 0.315 (0.288 - 0.341) | 0.268 (0.244 - 0.292) | 0.259 (0.235 - 0.282) |
| **2001** | 0.486 (0.454 - 0.519) | 0.254 (0.231 - 0.277) | 0.293 (0.268 - 0.318) | 0.312 (0.285 - 0.338) | 0.287 (0.262 - 0.312) | 0.264 (0.24 - 0.288) |
| **2002** | 0.425 (0.395 - 0.454) | 0.283 (0.258 - 0.308) | 0.334 (0.307 - 0.361) | 0.291 (0.266 - 0.316) | 0.26 (0.236 - 0.285) | 0.273 (0.249 - 0.297) |
| **2003** | 0.409 (0.38 - 0.437) | 0.291 (0.266 - 0.316) | 0.338 (0.31 - 0.365) | 0.314 (0.288 - 0.339) | 0.252 (0.228 - 0.275) | 0.284 (0.26 - 0.308) |
| **2004** | 0.354 (0.328 - 0.38) | 0.223 (0.203 - 0.244) | 0.262 (0.239 - 0.285) | 0.269 (0.245 - 0.293) | 0.244 (0.222 - 0.267) | 0.27 (0.246 - 0.294) |
| **2005** | 0.395 (0.367 - 0.423) | 0.23 (0.209 - 0.251) | 0.294 (0.27 - 0.318) | 0.282 (0.258 - 0.305) | 0.245 (0.222 - 0.267) | 0.254 (0.231 - 0.277) |
| **2006** | 0.34 (0.313 - 0.367) | 0.2 (0.179 - 0.22) | 0.23 (0.209 - 0.251) | 0.225 (0.204 - 0.246) | 0.213 (0.192 - 0.234) | 0.187 (0.168 - 0.206) |
| **2007** | 0.292 (0.269 - 0.316) | 0.193 (0.174 - 0.213) | 0.234 (0.212 - 0.255) | 0.218 (0.197 - 0.239) | 0.204 (0.184 - 0.224) | 0.173 (0.154 - 0.191) |
| **2008** | 0.263 (0.241 - 0.285) | 0.185 (0.165 - 0.204) | 0.262 (0.238 - 0.285) | 0.235 (0.214 - 0.257) | 0.218 (0.198 - 0.239) | 0.155 (0.138 - 0.171) |
| **2009** | 0.25 (0.229 - 0.272) | 0.184 (0.164 - 0.204) | 0.248 (0.225 - 0.27) | 0.204 (0.184 - 0.224) | 0.211 (0.191 - 0.231) | 0.166 (0.148 - 0.184) |
| **2010** | 0.258 (0.236 - 0.28) | 0.226 (0.205 - 0.247) | 0.25 (0.229 - 0.272) | 0.218 (0.198 - 0.238) | 0.228 (0.207 - 0.248) | 0.173 (0.154 - 0.191) |
| **2011** | 0.232 (0.212 - 0.253) | 0.181 (0.162 - 0.2) | 0.276 (0.253 - 0.299) | 0.214 (0.194 - 0.233) | 0.235 (0.215 - 0.256) | 0.16 (0.143 - 0.177) |
| **2012** | 0.264 (0.243 - 0.285) | 0.182 (0.164 - 0.199) | 0.287 (0.264 - 0.311) | 0.219 (0.199 - 0.239) | 0.189 (0.171 - 0.207) | 0.15 (0.134 - 0.166) |
| **2013** | 0.225 (0.206 - 0.245) | 0.168 (0.151 - 0.184) | 0.255 (0.233 - 0.277) | 0.218 (0.198 - 0.238) | 0.243 (0.223 - 0.264) | 0.166 (0.149 - 0.183) |
| **2014** | 0.259 (0.238 - 0.279) | 0.204 (0.186 - 0.223) | 0.283 (0.259 - 0.306) | 0.229 (0.21 - 0.249) | 0.253 (0.233 - 0.274) | 0.157 (0.14 - 0.173) |
| **2015** | 0.227 (0.208 - 0.246) | 0.207 (0.188 - 0.226) | 0.285 (0.263 - 0.307) | 0.216 (0.197 - 0.235) | 0.261 (0.24 - 0.281) | 0.171 (0.153 - 0.188) |
| **2016** | 0.225 (0.206 - 0.243) | 0.174 (0.157 - 0.191) | 0.273 (0.252 - 0.294) | 0.204 (0.186 - 0.222) | 0.251 (0.231 - 0.271) | 0.149 (0.134 - 0.165) |
| **2017** | 0.242 (0.221 - 0.263) | 0.172 (0.156 - 0.188) | 0.297 (0.275 - 0.319) | 0.195 (0.177 - 0.213) | 0.265 (0.245 - 0.286) | 0.175 (0.157 - 0.193) |
| **2018** | 0.238 (0.217 - 0.259) | 0.168 (0.151 - 0.185) | 0.276 (0.255 - 0.297) | 0.197 (0.179 - 0.215) | 0.265 (0.245 - 0.285) | 0.161 (0.144 - 0.177) |
| **2019** | 0.198 (0.182 - 0.215) | 0.179 (0.162 - 0.196) | 0.334 (0.311 - 0.357) | 0.187 (0.17 - 0.204) | 0.303 (0.28 - 0.326) | 0.165 (0.149 - 0.182) |
| **2020** | 0.272 (0.249 - 0.294) | 0.233 (0.214 - 0.253) | 0.367 (0.343 - 0.39) | 0.199 (0.183 - 0.216) | 0.343 (0.32 - 0.367) | 0.178 (0.162 - 0.195) |
| **2021** | 0.292 (0.27 - 0.315) | 0.257 (0.236 - 0.277) | 0.396 (0.372 - 0.42) | 0.232 (0.213 - 0.251) | 0.402 (0.375 - 0.428) | 0.187 (0.17 - 0.204) |
| **2022** | 0.285 (0.262 - 0.308) | 0.244 (0.224 - 0.264) | 0.386 (0.363 - 0.41) | 0.202 (0.185 - 0.219) | 0.342 (0.32 - 0.363) | 0.183 (0.166 - 0.199) |
| **2023** | 0.246 (0.226 - 0.266) | 0.205 (0.186 - 0.223) | 0.371 (0.348 - 0.395) | 0.183 (0.166 - 0.2) | 0.377 (0.352 - 0.401) | 0.189 (0.172 - 0.206) |
| **Mean** | **6.87 (6.74 - 7.01)** | **0.21 (0.19 - 0.23)** | **6.45 (6.32 - 6.58)** | **6.27 (6.14 - 6.40)** | **6.11 (5.98 - 6.23)** | **5.94 (5.81 - 6.06)** |

**Supplemental Table 6 Pulmonary Embolism related Forecast Age-Adjusted Mortality Rates, Stratified by Overall, Sex, Race, Age in Adults in the United States, 2024 to 2040**

| **Supplemental Table 6 Pulmonary Embolism related Forecast Age-Adjusted Mortality Rates, Stratified by Overall, Sex, Race, Age in Adults in the United States, 2024 to 2040** | | | |
| --- | --- | --- | --- |
| **Stratification** | **Year** | **Forecast Value (LCI - UCI)** | **Model** |
| Female | 2024 | 3.32 (3.18 - 3.48) | Prophet |
| Female | 2025 | 3.39 (3.22 - 3.56) | Prophet |
| Female | 2026 | 3.31 (3.16 - 3.49) | Prophet |
| Female | 2027 | 3.28 (3.12 - 3.45) | Prophet |
| Female | 2028 | 3.30 (3.14 - 3.47) | Prophet |
| Female | 2029 | 3.37 (3.19 - 3.57) | Prophet |
| Female | 2030 | 3.29 (3.11 - 3.49) | Prophet |
| Female | 2031 | 3.27 (3.07 - 3.47) | Prophet |
| Female | 2032 | 3.29 (3.08 - 3.51) | Prophet |
| Female | 2033 | 3.35 (3.11 - 3.61) | Prophet |
| Female | 2034 | 3.28 (3.01 - 3.56) | Prophet |
| Female | 2035 | 3.25 (2.96 - 3.53) | Prophet |
| Female | 2036 | 3.27 (2.97 - 3.58) | Prophet |
| Female | 2037 | 3.33 (3.00 - 3.69) | Prophet |
| Female | 2038 | 3.26 (2.89 - 3.64) | Prophet |
| Female | 2039 | 3.23 (2.83 - 3.63) | Prophet |
| Female | 2040 | 3.25 (2.83 - 3.69) | Prophet |
| Male | 2024 | 3.33 (3.00 - 3.69) | ARIMA |
| Male | 2025 | 3.25 (2.81 - 3.77) | ARIMA |
| Male | 2026 | 3.18 (2.66 - 3.81) | ARIMA |
| Male | 2027 | 3.11 (2.53 - 3.83) | ARIMA |
| Male | 2028 | 3.04 (2.41 - 3.83) | ARIMA |
| Male | 2029 | 2.97 (2.30 - 3.83) | ARIMA |
| Male | 2030 | 2.91 (2.21 - 3.82) | ARIMA |
| Male | 2031 | 2.84 (2.12 - 3.81) | ARIMA |
| Male | 2032 | 2.78 (2.03 - 3.79) | ARIMA |
| Male | 2033 | 2.72 (1.96 - 3.77) | ARIMA |
| Male | 2034 | 2.66 (1.88 - 3.75) | ARIMA |
| Male | 2035 | 2.60 (1.81 - 3.72) | ARIMA |
| Male | 2036 | 2.54 (1.75 - 3.69) | ARIMA |
| Male | 2037 | 2.48 (1.68 - 3.66) | ARIMA |
| Male | 2038 | 2.43 (1.62 - 3.63) | ARIMA |
| Male | 2039 | 2.37 (1.57 - 3.59) | ARIMA |
| Male | 2040 | 2.32 (1.51 - 3.56) | ARIMA |
| Overall | 2024 | 3.52 (3.33 - 3.72) | Prophet |
| Overall | 2025 | 3.58 (3.40 - 3.77) | Prophet |
| Overall | 2026 | 3.52 (3.35 - 3.71) | Prophet |
| Overall | 2027 | 3.51 (3.32 - 3.70) | Prophet |
| Overall | 2028 | 3.55 (3.37 - 3.76) | Prophet |
| Overall | 2029 | 3.61 (3.40 - 3.82) | Prophet |
| Overall | 2030 | 3.54 (3.34 - 3.77) | Prophet |
| Overall | 2031 | 3.53 (3.30 - 3.78) | Prophet |
| Overall | 2032 | 3.58 (3.34 - 3.85) | Prophet |
| Overall | 2033 | 3.63 (3.38 - 3.95) | Prophet |
| Overall | 2034 | 3.57 (3.30 - 3.89) | Prophet |
| Overall | 2035 | 3.56 (3.25 - 3.91) | Prophet |
| Overall | 2036 | 3.60 (3.26 - 4.00) | Prophet |
| Overall | 2037 | 3.66 (3.30 - 4.11) | Prophet |
| Overall | 2038 | 3.59 (3.21 - 4.07) | Prophet |
| Overall | 2039 | 3.58 (3.13 - 4.10) | Prophet |
| Overall | 2040 | 3.63 (3.18 - 4.20) | Prophet |
| Black or African American | 2024 | 6.86 (6.47 - 7.28) | Prophet |
| Black or African American | 2025 | 7.04 (6.63 - 7.46) | Prophet |
| Black or African American | 2026 | 6.96 (6.55 - 7.35) | Prophet |
| Black or African American | 2027 | 6.97 (6.56 - 7.39) | Prophet |
| Black or African American | 2028 | 7.06 (6.58 - 7.51) | Prophet |
| Black or African American | 2029 | 7.24 (6.76 - 7.71) | Prophet |
| Black or African American | 2030 | 7.16 (6.66 - 7.69) | Prophet |
| Black or African American | 2031 | 7.17 (6.58 - 7.72) | Prophet |
| Black or African American | 2032 | 7.27 (6.65 - 7.88) | Prophet |
| Black or African American | 2033 | 7.46 (6.76 - 8.14) | Prophet |
| Black or African American | 2034 | 7.37 (6.59 - 8.16) | Prophet |
| Black or African American | 2035 | 7.38 (6.57 - 8.25) | Prophet |
| Black or African American | 2036 | 7.49 (6.61 - 8.40) | Prophet |
| Black or African American | 2037 | 7.68 (6.59 - 8.70) | Prophet |
| Black or African American | 2038 | 7.59 (6.50 - 8.66) | Prophet |
| Black or African American | 2039 | 7.60 (6.42 - 8.78) | Prophet |
| Black or African American | 2040 | 7.71 (6.39 - 9.09) | Prophet |
| White | 2024 | 3.60 (3.41 - 3.77) | Prophet |
| White | 2025 | 3.64 (3.47 - 3.83) | Prophet |
| White | 2026 | 3.58 (3.40 - 3.78) | Prophet |
| White | 2027 | 3.58 (3.40 - 3.76) | Prophet |
| White | 2028 | 3.62 (3.44 - 3.84) | Prophet |
| White | 2029 | 3.67 (3.46 - 3.88) | Prophet |
| White | 2030 | 3.61 (3.40 - 3.85) | Prophet |
| White | 2031 | 3.60 (3.38 - 3.86) | Prophet |
| White | 2032 | 3.65 (3.40 - 3.93) | Prophet |
| White | 2033 | 3.69 (3.43 - 4.00) | Prophet |
| White | 2034 | 3.63 (3.35 - 3.97) | Prophet |
| White | 2035 | 3.63 (3.32 - 3.99) | Prophet |
| White | 2036 | 3.68 (3.35 - 4.06) | Prophet |
| White | 2037 | 3.72 (3.33 - 4.15) | Prophet |
| White | 2038 | 3.66 (3.27 - 4.16) | Prophet |
| White | 2039 | 3.66 (3.22 - 4.20) | Prophet |
| White | 2040 | 3.70 (3.24 - 4.31) | Prophet |
| Younger Adults (25-44) | 2024 | 0.99 (0.89 - 1.09) | Prophet |
| Younger Adults (25-44) | 2025 | 1.00 (0.90 - 1.10) | Prophet |
| Younger Adults (25-44) | 2026 | 1.00 (0.90 - 1.12) | Prophet |
| Younger Adults (25-44) | 2027 | 1.01 (0.91 - 1.11) | Prophet |
| Younger Adults (25-44) | 2028 | 1.01 (0.91 - 1.12) | Prophet |
| Younger Adults (25-44) | 2029 | 1.02 (0.91 - 1.13) | Prophet |
| Younger Adults (25-44) | 2030 | 1.03 (0.92 - 1.14) | Prophet |
| Younger Adults (25-44) | 2031 | 1.03 (0.93 - 1.15) | Prophet |
| Younger Adults (25-44) | 2032 | 1.03 (0.94 - 1.15) | Prophet |
| Younger Adults (25-44) | 2033 | 1.05 (0.94 - 1.17) | Prophet |
| Younger Adults (25-44) | 2034 | 1.05 (0.94 - 1.17) | Prophet |
| Younger Adults (25-44) | 2035 | 1.05 (0.95 - 1.17) | Prophet |
| Younger Adults (25-44) | 2036 | 1.06 (0.95 - 1.18) | Prophet |
| Younger Adults (25-44) | 2037 | 1.07 (0.97 - 1.19) | Prophet |
| Younger Adults (25-44) | 2038 | 1.07 (0.96 - 1.20) | Prophet |
| Younger Adults (25-44) | 2039 | 1.08 (0.97 - 1.19) | Prophet |
| Younger Adults (25-44) | 2040 | 1.08 (0.97 - 1.20) | Prophet |
| Middle-Aged Adults (45-64) | 2024 | 2.70 (2.38 - 3.05) | ARIMA |
| Middle-Aged Adults (45-64) | 2025 | 2.72 (2.32 - 3.19) | ARIMA |
| Middle-Aged Adults (45-64) | 2026 | 2.74 (2.29 - 3.27) | ARIMA |
| Middle-Aged Adults (45-64) | 2027 | 2.75 (2.28 - 3.33) | ARIMA |
| Middle-Aged Adults (45-64) | 2028 | 2.76 (2.27 - 3.36) | ARIMA |
| Middle-Aged Adults (45-64) | 2029 | 2.77 (2.27 - 3.39) | ARIMA |
| Middle-Aged Adults (45-64) | 2030 | 2.78 (2.27 - 3.41) | ARIMA |
| Middle-Aged Adults (45-64) | 2031 | 2.79 (2.27 - 3.42) | ARIMA |
| Middle-Aged Adults (45-64) | 2032 | 2.79 (2.27 - 3.43) | ARIMA |
| Middle-Aged Adults (45-64) | 2033 | 2.80 (2.27 - 3.44) | ARIMA |
| Middle-Aged Adults (45-64) | 2034 | 2.80 (2.27 - 3.45) | ARIMA |
| Middle-Aged Adults (45-64) | 2035 | 2.80 (2.27 - 3.45) | ARIMA |
| Middle-Aged Adults (45-64) | 2036 | 2.80 (2.28 - 3.46) | ARIMA |
| Middle-Aged Adults (45-64) | 2037 | 2.81 (2.28 - 3.46) | ARIMA |
| Middle-Aged Adults (45-64) | 2038 | 2.81 (2.28 - 3.46) | ARIMA |
| Middle-Aged Adults (45-64) | 2039 | 2.81 (2.28 - 3.46) | ARIMA |
| Middle-Aged Adults (45-64) | 2040 | 2.81 (2.28 - 3.46) | ARIMA |
| Older Adults (65+) | 2024 | 10.13 (9.68 - 10.67) | Prophet |
| Older Adults (65+) | 2025 | 10.30 (9.77 - 10.85) | Prophet |
| Older Adults (65+) | 2026 | 10.13 (9.64 - 10.72) | Prophet |
| Older Adults (65+) | 2027 | 10.00 (9.48 - 10.55) | Prophet |
| Older Adults (65+) | 2028 | 9.90 (9.34 - 10.44) | Prophet |
| Older Adults (65+) | 2029 | 10.07 (9.50 - 10.72) | Prophet |
| Older Adults (65+) | 2030 | 9.90 (9.30 - 10.55) | Prophet |
| Older Adults (65+) | 2031 | 9.77 (9.12 - 10.48) | Prophet |
| Older Adults (65+) | 2032 | 9.67 (8.94 - 10.41) | Prophet |
| Older Adults (65+) | 2033 | 9.84 (9.08 - 10.70) | Prophet |
| Older Adults (65+) | 2034 | 9.68 (8.80 - 10.56) | Prophet |
| Older Adults (65+) | 2035 | 9.55 (8.62 - 10.51) | Prophet |
| Older Adults (65+) | 2036 | 9.45 (8.46 - 10.47) | Prophet |
| Older Adults (65+) | 2037 | 9.61 (8.53 - 10.75) | Prophet |
| Older Adults (65+) | 2038 | 9.46 (8.25 - 10.69) | Prophet |
| Older Adults (65+) | 2039 | 9.33 (8.04 - 10.61) | Prophet |
| Older Adults (65+) | 2040 | 9.24 (7.88 - 10.57) | Prophet |
| LCI = Lower Confidence Interval |  |  |  |
| UCI = Upper confidence Interval |  |  |  |

**Supplemental Table 7 Pulmonary Embolism related Forecast Age-Adjusted Mortality Rates, Stratified by Comorbidities in Adults in the United States, 2024 to 2040**

| **Supplemental Table 7 Pulmonary Embolism related Forecast Age-Adjusted Mortality Rates, Stratified by Comorbidities in Adults in the United States, 2024 to 2040** | | | | | | |
| --- | --- | --- | --- | --- | --- | --- |
| **Year** | **PE + Coronary Artery Disease (ARIMA)** | **PE + Diabetes Mellitus (ARIMA)** | **PE + Hypertension (ARIMA)** | **PE + Heart Failure (ARIMA)** | **PE + COPD (ARIMA)** | **PE + Infection (ARIMA)** |
| 2024 | 0.25 (0.19 - 0.32) | 0.21 (0.16 - 0.26) | 0.37 (0.30 - 0.47) | 0.38 (0.31 - 0.48) | 0.19 (0.16 - 0.22) | 0.19 (0.16 - 0.23) |
| 2025 | 0.25 (0.17 - 0.35) | 0.21 (0.14 - 0.29) | 0.37 (0.27 - 0.51) | 0.40 (0.30 - 0.54) | 0.18 (0.15 - 0.22) | 0.19 (0.14 - 0.25) |
| 2026 | 0.25 (0.16 - 0.38) | 0.21 (0.13 - 0.32) | 0.37 (0.25 - 0.55) | 0.42 (0.28 - 0.61) | 0.18 (0.14 - 0.22) | 0.19 (0.14 - 0.26) |
| 2027 | 0.25 (0.15 - 0.41) | 0.21 (0.12 - 0.34) | 0.37 (0.24 - 0.58) | 0.43 (0.27 - 0.69) | 0.17 (0.14 - 0.22) | 0.19 (0.13 - 0.28) |
| 2028 | 0.25 (0.14 - 0.43) | 0.21 (0.12 - 0.36) | 0.37 (0.22 - 0.62) | 0.45 (0.26 - 0.79) | 0.17 (0.13 - 0.22) | 0.19 (0.12 - 0.29) |
| 2029 | 0.25 (0.13 - 0.45) | 0.21 (0.11 - 0.38) | 0.37 (0.21 - 0.65) | 0.47 (0.24 - 0.90) | 0.17 (0.13 - 0.22) | 0.19 (0.12 - 0.30) |
| 2030 | 0.25 (0.13 - 0.48) | 0.21 (0.10 - 0.40) | 0.37 (0.20 - 0.68) | 0.49 (0.23 - 1.03) | 0.16 (0.12 - 0.22) | 0.19 (0.11 - 0.31) |
| 2031 | 0.25 (0.12 - 0.50) | 0.21 (0.10 - 0.42) | 0.37 (0.20 - 0.70) | 0.51 (0.22 - 1.19) | 0.16 (0.12 - 0.22) | 0.19 (0.11 - 0.32) |
| 2032 | 0.25 (0.12 - 0.52) | 0.21 (0.10 - 0.44) | 0.37 (0.19 - 0.73) | 0.53 (0.20 - 1.38) | 0.15 (0.11 - 0.21) | 0.19 (0.11 - 0.33) |
| 2033 | 0.25 (0.11 - 0.54) | 0.21 (0.09 - 0.46) | 0.37 (0.18 - 0.76) | 0.55 (0.19 - 1.60) | 0.15 (0.11 - 0.21) | 0.19 (0.10 - 0.35) |
| 2034 | 0.25 (0.11 - 0.56) | 0.21 (0.09 - 0.48) | 0.37 (0.17 - 0.79) | 0.57 (0.17 - 1.87) | 0.15 (0.10 - 0.21) | 0.19 (0.10 - 0.36) |
| 2035 | 0.25 (0.10 - 0.58) | 0.21 (0.08 - 0.50) | 0.37 (0.17 - 0.81) | 0.59 (0.16 - 2.19) | 0.14 (0.10 - 0.21) | 0.19 (0.10 - 0.37) |
| 2036 | 0.25 (0.10 - 0.60) | 0.21 (0.08 - 0.51) | 0.37 (0.16 - 0.84) | 0.62 (0.15 - 2.57) | 0.14 (0.10 - 0.21) | 0.19 (0.10 - 0.38) |
| 2037 | 0.25 (0.10 - 0.63) | 0.21 (0.08 - 0.53) | 0.37 (0.16 - 0.87) | 0.64 (0.14 - 3.02) | 0.14 (0.09 - 0.20) | 0.19 (0.09 - 0.39) |
| 2038 | 0.25 (0.09 - 0.65) | 0.21 (0.08 - 0.55) | 0.37 (0.15 - 0.89) | 0.67 (0.12 - 3.57) | 0.14 (0.09 - 0.20) | 0.19 (0.09 - 0.40) |
| 2039 | 0.25 (0.09 - 0.67) | 0.21 (0.07 - 0.57) | 0.37 (0.15 - 0.92) | 0.69 (0.11 - 4.24) | 0.13 (0.09 - 0.20) | 0.19 (0.09 - 0.40) |
| 2040 | 0.25 (0.09 - 0.69) | 0.21 (0.07 - 0.59) | 0.37 (0.15 - 0.95) | 0.72 (0.10 - 5.04) | 0.13 (0.08 - 0.20) | 0.19 (0.09 - 0.41) |

**Supplemental Figure 1:** Pulmonary Embolism-Related AAMR Stratified By Age per 100,000 Population

 **Supplemental Figure 2.** Pulmonary Embolism-related AAMR Stratified by contributing causes of death per 100,000 population
